# Evaluation of right ventricle pulmonary artery coupling on right ventricular function in post operative tetralogy of Fallot patients underwent for pulmonary valve replacement

**DOI:** 10.1186/s13019-020-01281-1

**Published:** 2020-09-10

**Authors:** Bhushan Sandeep, Xin Huang, Yuan Li, Xiaowei Wang, Long Mao, Yue Kan, Dan Xiong, Ke Gao, Xiao Zongwei

**Affiliations:** 1 Department of Cardiothoracic surgery, Chengdu second People’s Hospital, Chengdu, 610017 Sichuan China; 2 Department of Anesthesiology, Chengdu second People’s Hospital Chengdu, Chengdu, 610017 Sichuan China

**Keywords:** Right ventricular pulmonary artery (RV-PA), Arterial elastance (Ea), End-systolic elastance (Ees), Cardiac magnetic resonance imaging (CMRI)

## Abstract

**Background:**

To evaluate RV-PA coupling in post operative TOF patients with ventricular dilatation underwent for PVR and investigate the correlation between ventricular functions measuring Ea/Emax ratio using cardio magnetic resonance and the effect of surgical type at primary repair of TOF on coupling.

**Method:**

RV-PA coupling was measured noninvasively by Ea/Emax ratio from CMRI and ECHO. From CMRI results the patients were divided in two groups, RV-PA coupling and RV-PA uncoupling. Ea/Emax ≤1 was considered for coupling patients and Ea/Emax > 1 for uncoupling patients.

**Results:**

Ninety patients were uncoupled (Ea/Emax: 1.55 ± 0.46) and 45 were coupled (Ea/Emax: 0.81 ± 0.15). Out of 75 TAP repaired patients 60 were uncoupled RV-PV. In addition, higher pro-BNP is an important factor for uncoupled RV-PV (*P* = 0.001). CMR evaluation for right ventricular function between uncoupling and coupling were RVEDVi (196.65 ± 63.57 vs. 154.28 ± 50.07, *P* = 0.001), RVESVi (121.19 ± 51.47 vs. 83.94 ± 20.43, *P* = 0.001), RVSVi (67.19 ± 19.87 vs. 106.31 ± 33.44, *P* = 0.001), and RVEF (40.90 ± 8.73 vs. 54.63 ± 4.76, *P* = 0.001). The increased RVEDVi, RVESVi and RVSVi and decreased RVEF have significant correlation with Ea/Emax. Ea/Emax was also found positively correlated with RVEDVi (*P = <* 0.05, *r* = 0.35), RVESVi (*P = <* 0.001, *r* = 0.41) and negatively correlated with RVSVi (*P = <* 0.05, *r* = 0.22) and RVEF (*P = <* 0.05, *r* = 0.78).

**Conclusions:**

Unfavorable RV-PA coupling is present in post operative TOF patients and it is affected by several factors. Our results explain a new concept of RV-PA interactions as a contributing mechanism for the observed decline in RV function.

## General introduction

Today, one of severe problem is an increasing number of TOF patients with right ventricular dysfunction which leads to ventricular dilatation and ventricular failure which in long term causes residual pulmonary valve regurgitation (PR) [1]. Cardiovascular magnetic resonance imaging (CMRI) techniques have visualized marked enlargement of the right ventricular dimensions in post-operative TOF patients with severe PR [6]. After a time period PR leads to develop RV dilatation which causes RV dysfunction, and it progresses into exercise intolerance, ventricular arrhythmias, and sudden death of patients [4]. Earlier studies mainly discuss the evaluation of RV function and dimensions. However, these pathophysiological changes are not done by RV alone; relatively, it is coupled to a highly compliant low pressure pulmonary arterial (PA) system which is known as right ventricular pulmonary artery (RV-PA) coupling. Consequently, RV chamber and myocardial adaptation cannot be considered independent from the PA system. RV-PA coupling is defined as the ratio of pulmonary artery elastance (an index of arterial load) and right ventricular end-systolic elastance (an index of contractility) [21]. RV-PA coupling occurs when there is maximum transfer of potential energy from one elastic chamber (the ventricle) to another (the pulmonary arterial system), and this process occurs when both of the elastance are equal. Therefore the ratio Ea/Emax is used as an index of RV-PA coupling. The ratio of end-systolic pressure divided by the stroke volume is defined as the effective pulmonary artery elastance (Ea) and it includes all components of total ventricular afterload including peripheral resistance, arterial compliance and characteristic impedance [19]. A load-independent parameter of myocardial contractility of ventricle is ventricular end-systolic elastance (Emax) and is typically calculated by invasive measurements with conductance catheters under various loading conditions and by non-invasive measurements by CMRI. The RV-RP coupling is calculated from the Ea/Emax ratio [18]. It has been indicated that normal RV functional adaptation to afterload is connected with maintenance of an Ea/Emax ratio of about 0.5, which allow for a flow output at a least amount of energy expense and the most favorable RV-PA coupling (ratio: 1) occurs when the RV and PA system both have equal elastance, which maximizes energy transfer and stroke work [15]. An adverse coupling between the RV and PA system with ineffective mechanical work production occurs when the Ea/Emax ratio increases by one and this is known as uncoupling [7]. The increased Ea/Emax ratio is a deciding factor in the progression of RV dysfunction and has currently been reported in post operative TOF patients. RV-PA uncoupling in post operative TOF patients underwent for PVR has not estimated in clinical studies. Our study is the first study regarding RV-PA coupling in post operative TOF patients underwent for pulmonary valve replacement (PVR). PVR is performed to overcome these adverse late effects with symptomatic beneficial outcome in remodeling of the right ventricle in addition with improved RV volumes and right ventricular function [8]. A marked RV remodeling occurred when the right ventricular end-diastolic volume becomes more than 150 ml/m^2^, and many researchers concluded that a cut-off level of 150 ml/m^2^ should be used as a clinical guide to recommend PVR [17]. As RV-PA coupling is not widely evaluated in repaired TOF patients, only one study shows that TOF patients have uncoupled RV-PA, our main objective is to evaluate RV-PA coupling in TOF patients and to investigate the correlation between ventricular functions using CMRI. Accordingly, using the CMRI technique, we used this coupling concept in post operative TOF patients with RV dilatation to reach a more meaningful understanding of RV performance to evaluate the role of the RV- PA coupling measuring Ea/Emax ratio, We also focused on either the surgical strategy at primary repair of TOF has any effect on RV-PA coupling.

## Patients and methods

### Study population

We retrospectively studied 135 post operative TOF patients admitted in Chengdu second people’s hospital for PVR between the time periods of January 2009 to February 2017. We assessed all these 135 patients and measured their age, weight, and blood pressure at the time of admission and HCT and Pro-BNP by blood routine test. For right ventricular evaluation we examined CMRI. Only post operative TOF patients underwent for PVR were selected. We excluded post operative TOF patients with other cardiac malformation, unilateral PA, and previous palliation.

#### Cardiovascular magnetic resonance imaging (CMRI)

CMRI examinations were performed in 45 post operative TOF patients who admitted for PVR on a 1.5-T magnetic resonance system (Magnetom Sonata) with a maximum gradient strength of 40 mT/m and a maximum slew rate of 200 mT/m/ms. No sedative medicines were applied during this process. The ventricular end-diastolic and end-systolic volumes, stroke volumes, and ejection fractions for both of the ventricles were estimated using the software dedicated for this. For all the patients the right ventricular and left ventricular examinations were performed, we calculated RVEDVi, RVESVi, RVSVi and RVEF for the right ventricle and LVEDVi, LVESVi, LVSVi and LVEF for the left ventricle. We used equation: Ea/Emax (CMR) = ESV/SV As discussed in previous literatures for defining coupling and uncoupling patients. ESV is ventricular end-systolic volume, SV is ventricular systolic volume.

### Statistical analysis

All continuous variables were tested for normality using the paired t-test for calculating *p*-values. The results are presented as the mean ± standard deviation. Comparisons of the Ea/Emax and other variables were performed using the t test for paired data. An unpaired, nonparametric Mann-Whitney U test was used to compare the patients who underwent a Transatrial/Transpulmonary approach and those with insertion of a transannular patch (TAP) at the primary repair. The categorical variables were analyzed by Pearson’s correlation. Analysis was performed using the SPSS version 25 and Graph Pad statistical software package, version 6.0 (Graph Pad, San Diego, Calif). *P* < .05 was considered statistically significant.

## Results

### Patient characteristics

The demographic data of these 135 patients are summarized in Table 1 below.

**Table 1:** Patient characteristics (*n* = 135)

Demographics	Parameters
Sex	
Male	75 (55%)
Female	60 (45%)
Weight (kg)	39.66 ± 18.43
Height (cm)	146.95 ± 23.40
Age at TOF Repair (Years)	8.52 ± 5.84
Age at PVR (Years)	19.88 ± 10.04
Surgery Type (TOF)	
1.TAP	75 (55%)
2.RVOT Patch	24 (18%)
3.Transatrial/Transpulmonary	21 (16%)
4.BT SHUNT	0
5.RV-PA conduit	15 (11%)
PVR	135
Surgical Approach	90 (67%)
Trans Catheter	45 (33%)
Hb	115 ± 10.72
HCT	0.36 ± 0.03
QRS Duration	147.6 ± 20.51
Blood Pressure	
Systolic (mm Hg)	106.6 ± 11.4
Diastolic (mm Hg)	68.8 ± 12.4
Pro-BNP	211.1 ± 65.1

#### Clinical outcome

Out of 135 patients RV-RP uncoupling was identified in 90 patients (66.7%), 45 patients have coupled RV-RP on basis of CMRI examinations. Both groups were compared in terms of gender, weight, height, age at TOF repair, age at PVR, type of TOF repair, method of PVR, Hb, Hct, QRS duration, blood pressure and Pro-BNP were measured in both coupled and uncoupled group of patients. (Table 2) In our study males predominates females (55%). In our study the gender, weight, height, age at TOF and age at PVR, Hb, HCT and blood pressure did not appear as a significant factor for RV-RP uncoupling. We measured Pro-BNP just before PVR in all patients and Pro-BNP emerges as a strong predictor for uncoupling with a significant *p*-vale 0.001.

**Table 2:** Results of clinical outcome

Variables	Coupling pts.(45)	Uncoupling pts.(90)	*P*-value
Sex			
Male	21 (28%)	54 (72%)	0.396
Female	24 (40%)	36 (60%)	
Weight	36.66 ± 14.66	44.74 ± 12.93	0.065
Height	142.20 ± 21.33	153.46 ± 15.63	0.50
Age at TOF repair(Y)	6.70 ± 6.42	6.31 ± 4.92	0.822
Age at PVR (Y)	19.70 ± 6.92	19.71 ± 9.37	0.997
Surgery type TOF			
1. TAP	15	60	< 0.001
2. RVOT	12	12	
3. TA/TP	12	9	
4. BT- stunt	0	0	
5. RV-PA conduit	6	9	
PVR			
Surgical approach	30	60	
Catheter method	15	30	
Hb	114.46 ± 12.00	115.88 ± 9.98	0.468
Hct	0.36 ± 0.04	0.35 ± 0.03	0.106
Qrs duration	149 ± 23.19	150.36 ± 16.60	0.696
Blood pressure			
Systolic	106 ± 11.21	110.44 ± 10.28	0.023
Diastolic	60.06 ± 11.14	73.64 ± 10.45	0.001
Pro-BNP	171.33 ± 70.25	237.76 ± 64.29	0.001

*TAP* Trans annular patch, *RVOT* Right ventricle outflow tract, *TA/TP* Trans atrial/Trans pulmonary, *RV-PA* Right ventricle-Pulmonary artery, *HB* Hemoglobin, *Hct* Hematocrit

#### CMRI correlations between coupling and uncoupling

The hemodynamic data measured at each state are presented in Table 3. In uncoupling patients, the RV end-systolic volume was greater than coupling patients with a significant *p*-value0.001. (Table-3). The hemodynamic data also shows a greater indexed RV end systolic volume in uncoupling patients compare with coupling patients with a significant *p*-value 0.001. A reduced mean RVEF was found in uncoupling patients than coupling patients with a significant p-value 0.001. Most of LV hemodynamic shows no or less change between coupling and uncoupling patients except LV ejection fraction with a significant *p*-value of 0.001, so the LV hemodynamic shows a weak correlation with RV-PA coupling. Ea/Emax ratio was 0.81 ± 0.15 in coupling patients which was almost double in uncoupling patients which was 1.55 ± 0.46 with a significant *p*-value of< 0.001.

**Table 3:** MRI analysis for coupling and uncoupling

Variables	Coupling	Uncoupling	*P*-value
No of Pts	45	90	
RVEDVi (ml/m^2^)	154.28 ± 50.07	196.65 ± 63.57	0.001*
RVESVi (ml/m^2^)	83.94 ± 20.43	121.19 ± 51.47	0.001*
RVSVi (ml/m^2^)	106.31 ± 33.44	67.19 ± 19.87	0.001*
RVEF (%)	54.63 ± 4.76	40.90 ± 8.73	0.001*
LVEDVi (ml/m^2^)	92.67 ± 21.10	95.98 ± 65.78	0.743
LVESVi (ml/m^2^)	37.75 ± 8.35	44.79 ± 15.25	0.005
LVSVi (ml/m^2^)	54.16 ± 14.64	49.08 ± 17.79	0.100
LVEF (%)	59.19 ± 4.47	52.63 ± 9.32	0.001*
Ea/Emax	0.81 ± 0.15	1.55 ± 0.46	0.001*

#### Factors affecting RV-PA coupling

##### Pro-BNP and Pearson’s correlation

**Fig. 1:**
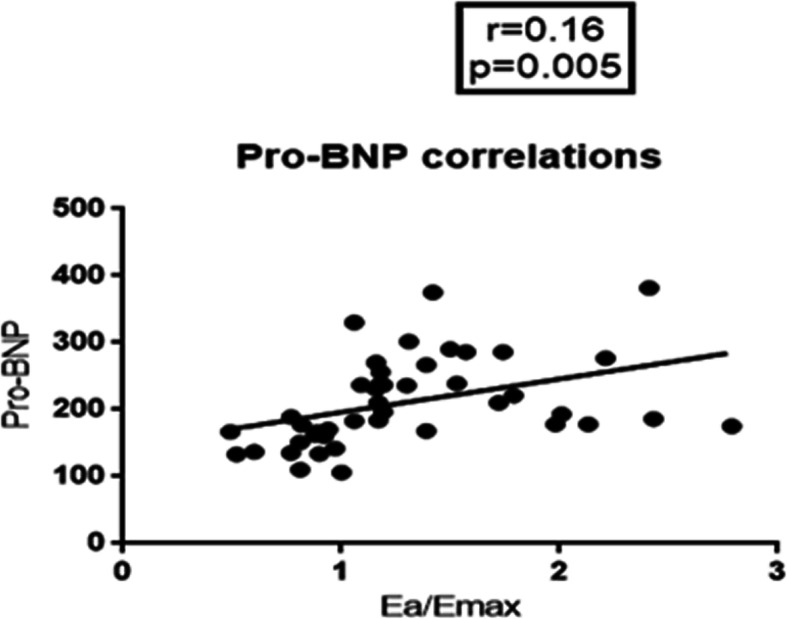
Correlation between Ea/Emax and Pro-BNP

When we correlated Pro-BNP with Ea/Emax by Pearson’s correlation it shows a linear correlation with r value 0.16 and *p*-value 0.005 (Fig. 1).

##### Right ventricular volumetric correlations

**Fig. 2:**
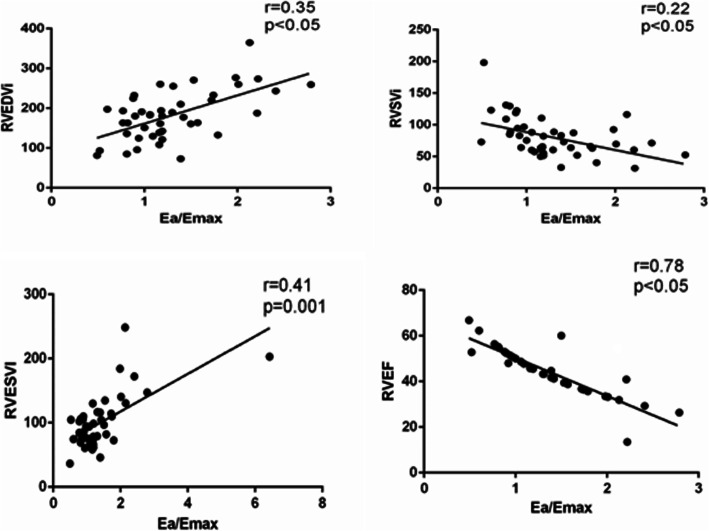
Relationship between Ea/Emax and indexed right ventricle end diastolic volume (RVEDVi), indexed right ventricle end systolic volume (RVESVi) right ventricular ejection fraction (RVEF) and indexed RV systolic volume

The Ea/Emax showed a significant relationship with RVEDVi, RVESVi, RVSVi and RVEF when we correlate these data with Pearson’s correlation equation. The Ea/Emax shown a positive linear relationship with CMRI-derived RVEDVi with r = 0.35 and *p*-valve < 0.005. Correlation with RVESVi with Ea/Emax was positive linear expression with r value 0.41 and a significant *p*-valve of 0.001. The Ea/Emax showed a significant inverse relationship with RVSVi and RVEF. The r value for RVSVi was 0.22 and p-valve was < 0.05 which was found significant. For RVEF the r value was 0.78 and a significant *p*-value of < 0.05. An increase in Ea/Emax thus describes the relative uncoupling of the RV-PA interaction, where afterload exceeds the ability of the RV to adapt. Thus, Ea/Emax is inversely related to RV ejection fraction (Fig. 2).

#### Effect of surgical strategy

**Fig. 3:**
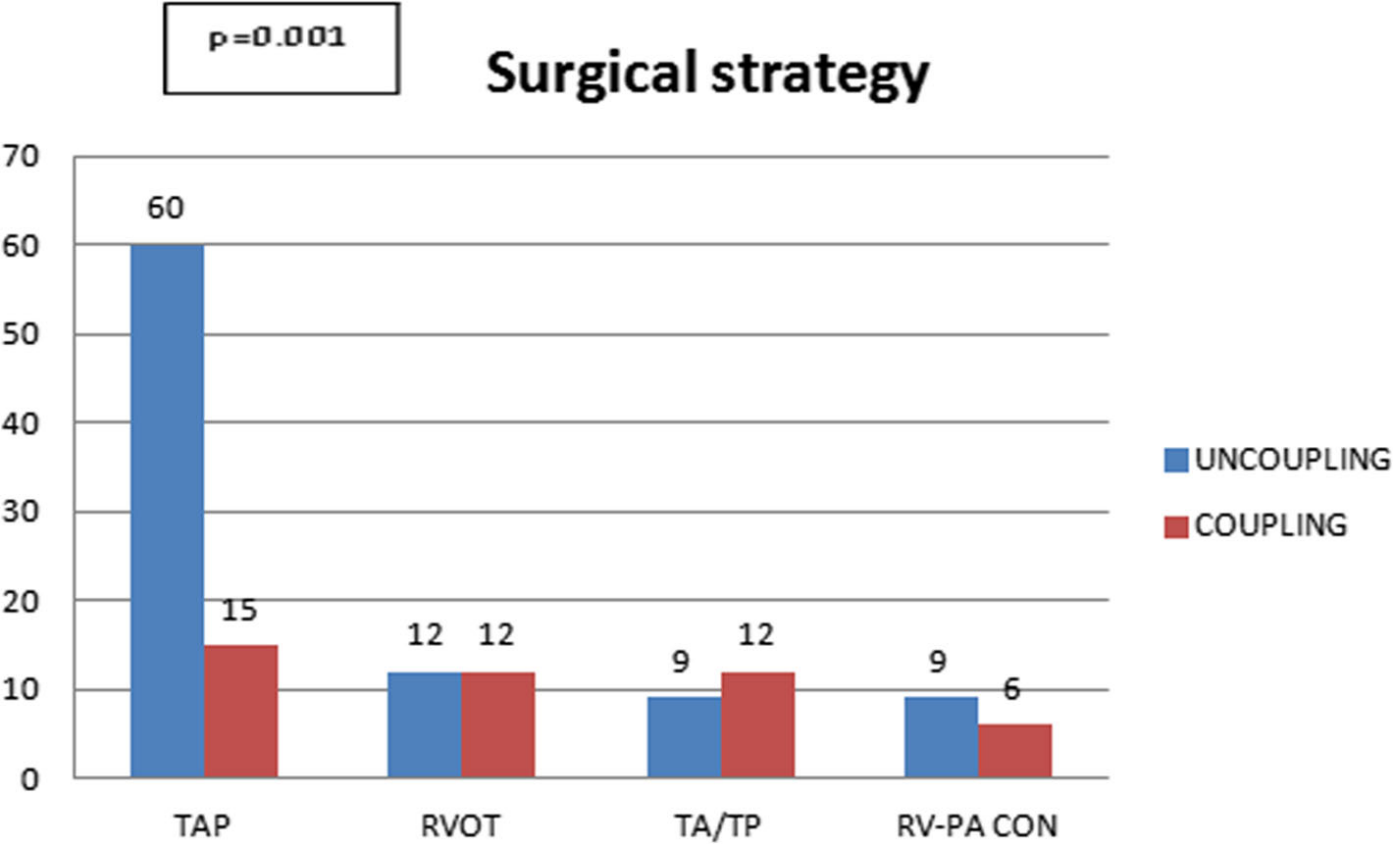
Surgical strategy in TOF repair

To evaluate whether the surgical procedure at the primary repair at TOF affects RV-PA coupling, we compared the 75 patients who had TAP repair with 18 patients who have RVOT repair, 21 patients with the Transatrial/Transpulmonary (TA/TP) repair, and 15 patients with RV-PA conduit repair. Out of 75 TAP repaired patients 60 patients were uncoupled, in 24 RVOT patients 12 patients were uncoupled, in 21 TA/TP patients 9 patients were uncoupled and in 15 patients with RV-PA conduit 9 patients were uncoupled (Fig. 3). There was no significant difference of age, weight, and height in TAP, RVOT, RV-PA conduit and TA/TP group of patients.

#### RV-PA coupling and its correlation in TOF

**Fig. 4:**
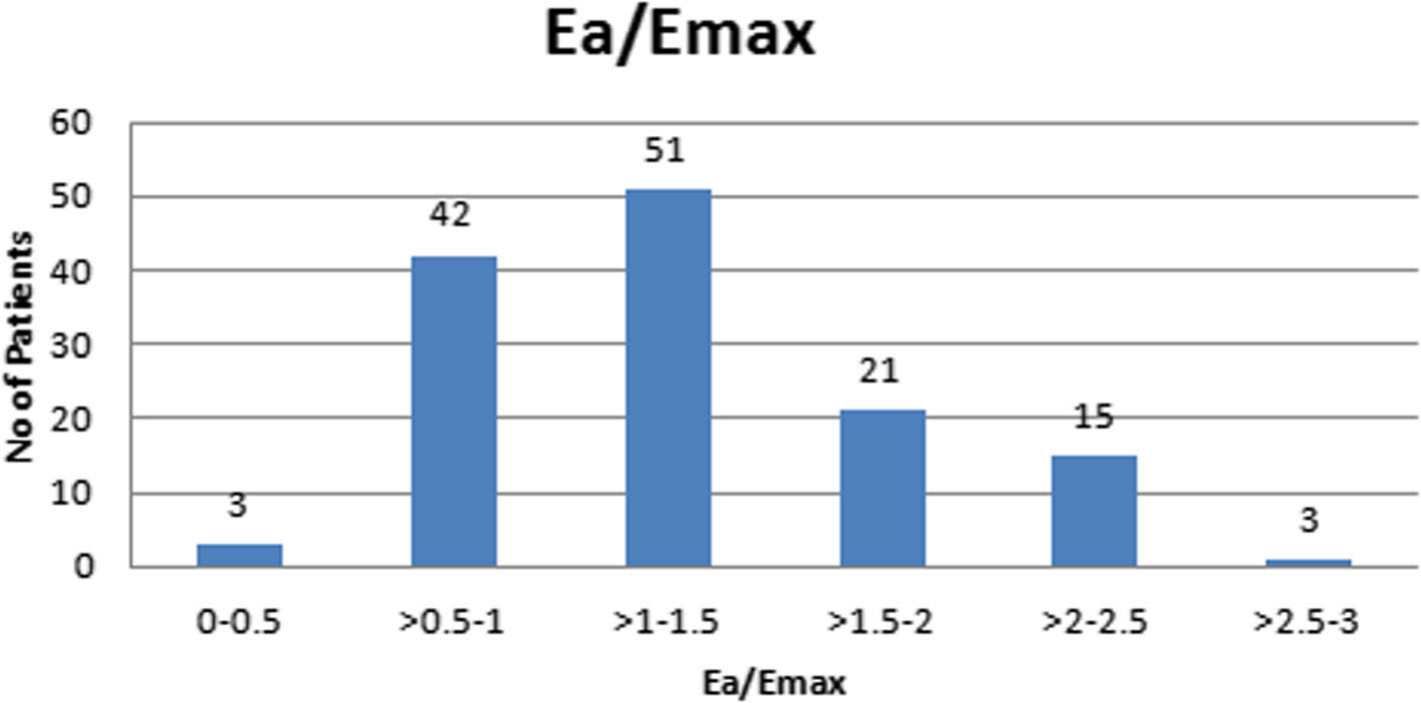
Distribution of patients on Ea/Emax value

From CMRI data we calculated Ea/Emax ratio for all 135 patients and from Ea/Emax ratio the patients were divided in two groups coupling and uncoupling respectively. Ea/Emax ≤1 was considered for coupling patients and Ea/Emax > 1 for uncoupling patients. On the basis of Ea/Emax ration the patient’s distribution is shown in (Fig. 4) below. For coupling patients 3 patients Ea/Emax was between 0 and 0.5 and 42 patients ranges between Ea/Emax > 0.5–1. In uncoupling patients majority of patients (*n* = 51) the Ea/Emax ration was between > 1–1.15. Twenty-one patient’s Ea/Emax ranges between > 1.5–2 and 15 patients were between Ea/Emax > 2–2.5. Only 3 uncoupled patient Ea/Emax was > 2.5.

### Impact of PVR on ventricular performance (follow up)

#### CMRI

After six months of PVR all 135 patients were followed up with CMRI. We compared 90 uncoupled RV-RP patients with their previous CMRI data to check out either their ventricular performance have been improved or not. Their follow up MRI data is below in the Table 4.

**Table 4:** CMRI data before and after PVR

CMRI variables	Before PVR (*N* = 90)	After PVR (*N* = 90)	*P*-value
RVEDVi (ml/m^2^)	221.50 ± 75.32	137.21 ± 8.91	0.001*
RVESVi (ml/m^2^)	146.62 ± 56.19	70.5 ± 10.04	0.001*
RVSVi (ml/m^2^)	101.05 ± 21.01	79.06 ± 9.38	0.001*
RVEF (%)	45.01 ± 7.48	54.35 ± 2.35	0.001*
Ea/Emax	1.43 ± 0.36	0.88 ± 0.05	0.001*

## Discussion

RV-PA uncoupling exists in a significant number of patients. When the Ea/Emax ratio increases by one an unfavorable uncoupling between the RV-PA vascular system occurs, which leads to an unproductive mechanical work production. This uncoupling between the RV and PA is a significant determinant in the development of RV dilatation which causes RV failure and has recently been reported in post operative TOF patients. We described a new noninvasive approach for the quantification of RV-PA coupling with simple measures derived from CMRI. A previous study of six patients with pulmonary hypertension using a CMRI-compatible catheter validated an invasive RV-PA coupling evaluation using CMRI-derived volumetric data [20].

Our study is the first description of RV-PA coupling in a large number of post operative TOF patients underwent for PVR with PR of a wide range of severity. Our findings support the theoretical outcomes and limited experimental observations regarding Ea/Emax. We found median Ea/Emax values of 0.81 ± 0.15 mm in coupling patients and 1.55 ± 0.46 in uncoupling patients. This is a good outcome with previously reported non invasive calculations. At most favorable coupling (ratio: 1) RV is capable of generating a maximum flow with minimum energy loss. In turn, maximum efficiency occurs when stroke work is maximum and oxygen consumption is minimum [21]. The RV circulation is nearly matched optimally in normal conditions [2]. In this condition, the RV works at maximum efficiency and sub-maximal stroke work (Ea < Emax). During acute ventricular dilatation and, as reported in several investigation, in earlier phases of ventricular failure, contractility increases to match with increased load and to maintain the SV, maintaining optimal coupling (Ea/Emax) at the cost of suboptimal mechanical efficiency (which causes increased oxygen demand) [7]. In this course, the RV fails as a pump with a decreased Emax and a larger increase in Ea/Emax which indicates insufficient coupling and reduced myocardial efficiency. PVR has been proved in improvement of ventricular function, and dilatation, stabilizing the QRS duration [21]. But there is also contradiction as a previous study supported that if RV function is significantly impaired it is unlikely to recover after PVR [16].

In our study RV-PA coupling is impaired in 90 patients, reflects maladaptive effects of the PA system on chronic volumetric loading in the presence of RV-retentive contractions. Additionally, we found that the surgical procedures used at the TOF repair has a major effect on the RV-PA coupling, the patients who received a TAP repair have significantly uncoupled RV-PA than patients with Transatrial TOF repair. Many previous studies demonstrated the mechanisms for decline in RV function and performance in patients after TOF repair. These studies mainly focus on the dimension and function of the right ventricle; but, in previous studies RV-PA coupling has never been studied systematically in post operative TOF patients. Normally the absolute stroke volume is increased to maintain sufficient cardiac output when longstanding severe PR is present after TOF repair. This condition leads to subsequent chronic volume overload of RV and proximal PA systems. After a time period, a progressive increase in the size and capacitance of the main PA is frequently observed in repaired TOF patients after several years. The situation is more complicated in TOF patients because the highly compliant PA system reduces the RV postload but due to the elastic retraction of the PA early in diastole it also worsens the PR in the absence of a functional pulmonary valve [12]. When the PA loses its elasticity it mainly depends on its original size and tissue structure resulting in a subsequent increase in stiffness and the RV pulsatile afterload [9]. Therefore one might wonder whether some form of vascular adaptation of the PA occurs in chronic volume overload to maintain the optimal coupling between the RV and the vascular system. Recently it has been reported that abnormal elastic tissue architecture and increased degree of moderate fibrosis (destruction of elastic tissue) and fibrosis in the pulmonary trunk of patients with TOF may affect mechanical wall properties [3]. Supporting a major role for major pulmonary arteries studies using four-dimensional CMRI velocity maps shows an increased wall shear stress in major pulmonary trunks in patients with TOF [5]. Considering that previously explained aortic histological malformations that also influence left ventricular function in patients with post operative TOF [13, 14]. The stroke volume output and end-systolic pressure ratio known as effective Ea represent valid estimates of systolic afterload in the systemic circulation and PA systems [10]. This parameter contains all the components of the vessel load including PA compliance which is necessary for sufficient RV-PA coupling [16]. Because Ea is mainly affected by PR these indices show a significant negative correlation.

We also found a significant negative correlation between Ea / Emax and RV ejection fraction indicating that RV function is not only affected by volume overload but also directly affected by pulsatile afterload. This finding suggests that only the PR assessment may not reflect the total hemodynamic burden of the RV following TOF repair. Depending on the size of the annulus and the PA system surgical treatment involves conventional TOF repair via the transpulmonary approach or through a ventricular incision usually using an implanted TAP [11]. Under positive inotropic stimulation the uncoupling of RV-PA observed in the TAP group was a result of a significant increase in Ea and a slow increase in Emax compared with Transatrial repair group. The findings indicate an unadaptable vascular response to increasing stroke volume followed by an increase in RV pulsatility afterload and may reflect the non-shrinkable patch material implanted at the RV-PA junction directly affecting ventricular performance and pulmonary vasculature characteristic.

### Study limitations

Our study population is small and in particular the subgroup analysis included only 135 patients. Although we were able to exclude this as a confounding factor in our study, the effect of aging after PVR on Ea/Emax remains unknown. The present study was mainly designed to perform a retrospective analysis to reveal causes of uncoupling after repair of TOF and to compare the sequele of different surgical approaches and CMRI, examinations. We can only speculate on the reasons by which they have occurred and their implications for the patients’ clinical condition and outcomes. Additional studies are necessary to evaluate the effect of Ea, Emax, and Ea/Emax and CMRI during follow up years and their outcome.

## Conclusions

RV-PA uncoupling is prevalent in repaired TOF patients and is affected by several factors. The degree of uncoupling is associated with patients RV dysfunction. By evaluation of the RV-PA coupling, the results of our study provide new insights into the RV-PA response to ventricular dilatation which can help in additional understanding for the decline of RV performance. Also the results explains the emerging role of RV-PA interactions as a contributing mechanism for the observed decline in RV function and impairment in post operative TOF patients underwent for PVR.

## Data Availability

Not applicable, Please contact author for data requests.

## Abbreviations

CMRI: Cardiac magnetic resonance imaging
Ea: Arterial elastance
Ees: End-systolic Elastance
PVR: Pulmonary valve replacement
PR: Pulmonary valve regurgitation
RV-PA: Right ventricle pulmonary artery
RV: Right ventricle
TOF: Tetralogy of Fallot
TAP: Transannular patch
